# Integrating Structures and Biology: Cellular and Molecular Interactions with Functionally Graded Spinal Cage Designs

**DOI:** 10.3390/ijms27104531

**Published:** 2026-05-18

**Authors:** Yuen Ho Cheng, Amy Libing Fu, Jessica Gaff, Gianluca Vadala, Amit Jain, Javad Tavakoli

**Affiliations:** 1 Department of Biomedical Engineering, School of Engineering, RMIT University, Melbourne 3000, Australia; s3875364@student.rmit.edu.au (Y.H.C.); amy.libing.fu@rmit.edu.au (A.L.F.); 2 Neurospine Institute, Murdoch, Perth 6150, Australia; jessica.gaff@neurospineinstitute.com.au; 3 Laboratory of Regenerative Orthopaedics, Operative Research Unit of Orthopaedic and Trauma Surgery, Fondazione Policlinico Universitario Campus Bio-Medico, 00128 Rome, Italy; g.vadala@gmail.com; 4 Department of Orthopaedic Surgery, Johns Hopkins University, Baltimore, MD 21287, USA; amitjain@jhmi.edu

**Keywords:** functionally graded materials, spinal fusion, interbody cage, additive manufacturing, osseointegration, mechanobiology, bone–implant interface

## Abstract

Interbody fusion cages are widely used to restore spinal stability, yet conventional designs often exhibit mechanical mismatch and limited biological integration. Functionally graded spinal cages incorporate spatial variations in composition and structure to better align mechanical properties with the surrounding bone environment. Although these designs have been extensively studied from an engineering perspective, their biological implications remain less clearly defined. This review examines how graded material composition, surface characteristics, porosity, and lattice architecture are associated with cellular and molecular responses relevant to bone regeneration. Reported biological responses include protein adsorption, immune modulation, angiogenesis, and osteogenic differentiation. Evidence from orthopaedic implants and tissue engineering systems suggests that such design features may influence mechanobiological pathways; however, direct experimental validation in spinal applications remains limited. Previous reviews primarily focus on material properties or mechanical performance of functionally graded spinal cages. This review presents a structured design-to-biology perspective linking graded implant features with biological responses relevant to spinal fusion. By integrating findings across biomaterials, mechanobiology, and implant design, this review presents a structured design-to-biology perspective and highlights current evidence, translational limitations, and key knowledge gaps in the field. Functionally graded spinal cages represent a promising but still evolving strategy, and further spine-specific mechanobiological and clinical studies are required to establish their impact on fusion outcomes.

## 1. Introduction

Low back pain represents a major global health burden, with lumbar degenerative disc disease contributing substantially to chronic disability. When conservative treatments fail, spinal fusion is often employed to restore spinal stability and alleviate pain [1,2,3]. This procedure typically involves the placement of an interbody fusion cage to provide mechanical support and facilitate bone formation between adjacent vertebral bodies [4]. Although current cage designs can achieve initial stability, clinical complications such as implant subsidence and delayed fusion remain prevalent [5,6,7]. Recent advances in additive manufacturing have enabled the development of functionally graded material (FGM) spinal cages, in which material composition and structural properties vary spatially across the implant [8,9,10]. FGM spinal cages exhibit gradual changes in composition or structure over their volume, permitting the mechanical properties of the implant to match those of native bone [11,12]. These gradual changes in properties may enhance load transfer and reduce stress shielding while enabling higher biomimetic interactions at the bone-implant interface [13]. By tuning porosity, stiffness, and surface architecture, FGM cages aim to balance mechanical performance with biological requirements for spinal fusion, potentially reducing post-surgical complications [14,15,16,17,18]. Spine-related and orthopaedic biomaterial studies have reported associations between parameters such as pore size, gradient orientation, and surface roughness and changes in cell adhesion, proliferation, and differentiation [19]. Mechanobiological models and experimental studies further suggest that these structural features may influence cellular signalling pathways involved in mechanotransduction and inflammatory regulation [20]. In addition, studies involving FGM spinal cages, orthopaedic implants and tissue engineering systems have associated spatial variations in material composition with changes in osteogenic activity and inflammatory responses [21,22]. However, many of these biological mechanisms remain incompletely validated in spine-specific FGM models, and the strength of evidence varies across experimental systems. As a result, the relationships between graded material and structural characteristics and the downstream biological responses relevant to spinal fusion remain fragmented across the current literature. These relationships are often discussed without consistent integration between engineering and biological perspectives.

An additional challenge lies in the nature of the available evidence. Much of the current understanding of cell–material interactions in FGM systems is derived from orthopaedic implants, dental devices, and tissue engineering scaffolds. Although these systems share fundamental biological mechanisms, spine-specific models remain limited, and their direct applicability to spinal fusion cages may be influenced by differences in anatomical environment, loading conditions, and clinical context. Mechanistic interpretations are therefore frequently extrapolated across related research domains, although the limitations of these comparisons are not always explicitly addressed. Consequently, biological effects reported in one experimental or clinical context may not be directly transferable to spinal fusion applications.

The present review aims to synthesise evidence across biomaterials, mechanobiology, and implant design. It examines how graded material composition, surface properties, porosity, and lattice architecture are associated with biological responses relevant to spinal fusion. Rather than providing a descriptive overview, this review adopts a structured design-to-biology perspective. It links key design parameters to cellular and molecular processes. At the same time, it explicitly acknowledges current evidence gaps and translational constraints. Attention is given to immune regulation, vascularisation, and mechanotransduction, areas that remain underexplored in spine-specific studies. By integrating insights from multiple domains while maintaining a critical perspective, the review aims to provide a more coherent foundation. It seeks to clarify the biological implications of functionally graded spinal cages and aims to inform the development of more effective and biologically responsive implant designs.

## 2. Methodology

A structured literature review was conducted to identify studies published between 2015 and 2025 relevant to functionally graded spinal cages. Searches were performed in PubMed, Scopus, and Web of Science. Keywords were selected to reflect both material design parameters and biological performance outcomes of spinal implants. Search terms included “spinal cages,” “functionally graded materials,” and “functionally graded spinal cages,” together with design-related terms such as “porosity,” “lattice size,” “composition,” “geometry,” “surface roughness,” and “mechanical properties.” Additional terms were incorporated to address biological responses, including “cell adhesion,” “cell proliferation and differentiation,” “mechanotransduction,” “cytokine expression,” “gene expression,” “protein secretion,” and “signalling pathways”.

Studies were included if they reported on material design features or biological responses relevant to spinal implants, orthopaedic devices, or bone tissue engineering scaffolds. Studies focusing exclusively on mechanical performance without biological evaluation were excluded. Additional articles were identified through manual screening of reference lists. Literature was screened based on relevance to graded spinal cage design and biological performance. Bibliographies of selected articles were also reviewed to identify further eligible publications. Given the limited availability of spine-specific studies on functionally graded cages, the current review also incorporates evidence from orthopaedic implants and bone tissue engineering scaffolds. These systems share fundamental biological processes, including bone remodelling, immune response, and mechanotransduction. Mechanistic relationships between structural design parameters and biological outcomes were therefore interpreted across related domains. While this approach enables a more comprehensive synthesis, it also highlights the current lack of direct experimental validation in spinal applications. Direct translation to spinal fusion applications therefore requires careful interpretation due to differences in anatomical environment, loading conditions, and clinical context. The extrapolation of findings across disciplines is based on shared biological principles but remains subject to inherent limitations, and the strength of these interpretations depends on the consistency of evidence across multiple model systems. In addition, variability in material composition, manufacturing methods, experimental conditions, and outcome measures makes direct comparison across studies challenging. Furthermore, while the references cited are generally current, some foundational studies have been retained to provide historical and mechanistic context where more recent data remain limited. As a result, the relationships described in the current review should be interpreted as indicative trends supported by heterogeneous evidence, rather than definitive conclusions.

## 3. Functionally Graded Spinal Cages

FGM spinal cages can be broadly classified based on design principles that address both engineering requirements and intended biological function (Table 1). Together, these principles describe how material composition, internal architecture, mechanical behaviour, surface bioactivity, and manufacturing strategy are integrated to more closely replicate the native vertebral environment.

**Figure 1:**
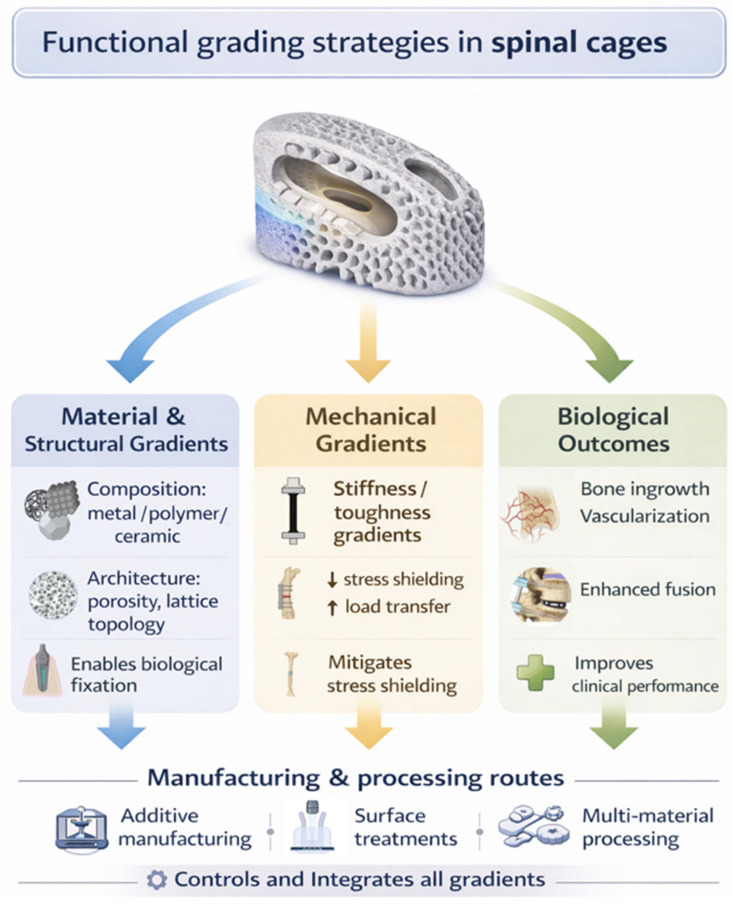
Conceptual framework for the classification of functionally graded material (FGM) spinal cages, illustrating how material and structural gradients, mechanical property gradients, and biologically driven outcomes are integrated through advanced manufacturing and processing routes to enhance load transfer, bone ingrowth, and fusion performance.

At the material level, FGM spinal cages frequently incorporate transitions from metals to ceramics, such as titanium-hydroxyapatite systems, to combine mechanical robustness with osteoconductive bioactivity. Alternatively, polymer–ceramic systems (e.g., PEEK–βTCP) are used to preserve radiolucency while improving biological interactions at the bone–implant interface. Although material choices vary, many designs follow a similar concept, consisting of a mechanically stable, relatively inert core that transitions toward a more biologically active outer region (Figure 1) [23]. Beyond composition, functional grading is commonly achieved through spatial variations in porosity and lattice architecture [24]. Common design approaches include a dense central region for structural support, with porosity increasing toward the implant surface to promote bone ingrowth and vascularisation. Similarly, lattice parameters such as pore size, strut thickness, and unit-cell geometry are often graded along the implant volume to reconcile mechanical compliance with biological compatibility [13,14]. Surface modifications, such as gradients in roughness or the incorporation of nano-topographical features, may also influence cellular attachment, proliferation, and differentiation.

From a mechanical standpoint, FGM designs allow implant stiffness to more closely match that of the surrounding vertebral bone. Gradual modulation of stiffness can reduce stress shielding and support more physiological load transfer [5]. In some designs, gradients in hardness or toughness are also introduced to enhance fatigue resistance and support stable fusion across the bone–implant interface. Additive manufacturing techniques, particularly selective laser melting (SLM) and electron beam melting (EBM), allow precise spatial control over geometry and porosity [8]. In some cases, a single material is used while structural parameters are varied; in others, multiple materials with distinct properties are combined within a single construct. Complementary approaches, including powder metallurgy and surface coating techniques such as plasma spraying or sol–gel processing, are often employed to introduce compositional gradients, particularly at the implant surface [25].

Finally, FGM spinal cages may also be categorised according to anatomical location and clinical application. Cervical and lumbar cages differ substantially in size, design, and load-bearing demands, necessitating distinct gradient strategies. Design priorities also vary with application, with some cages emphasising mechanical stability in high-load regions, whereas others focus on reproducing the local biological and mechanical environment to support fusion [9,19,26].

## 4. Biological Impact of Functionally Graded Spinal Cages

The biological effects of functionally graded spinal cages are discussed according to three primary design domains: (i) material gradients, which influence surface chemistry and protein-mediated interactions; (ii) structural gradients, including porosity and lattice architecture, which regulate cell behaviour and mass transport; and (iii) mechanical gradients, which govern load transfer and mechanotransduction. While these domains are conceptually distinct, they are inherently interrelated and may act synergistically to influence biological responses.

### 4.1. Material Gradient and Composition

**Figure 2:**
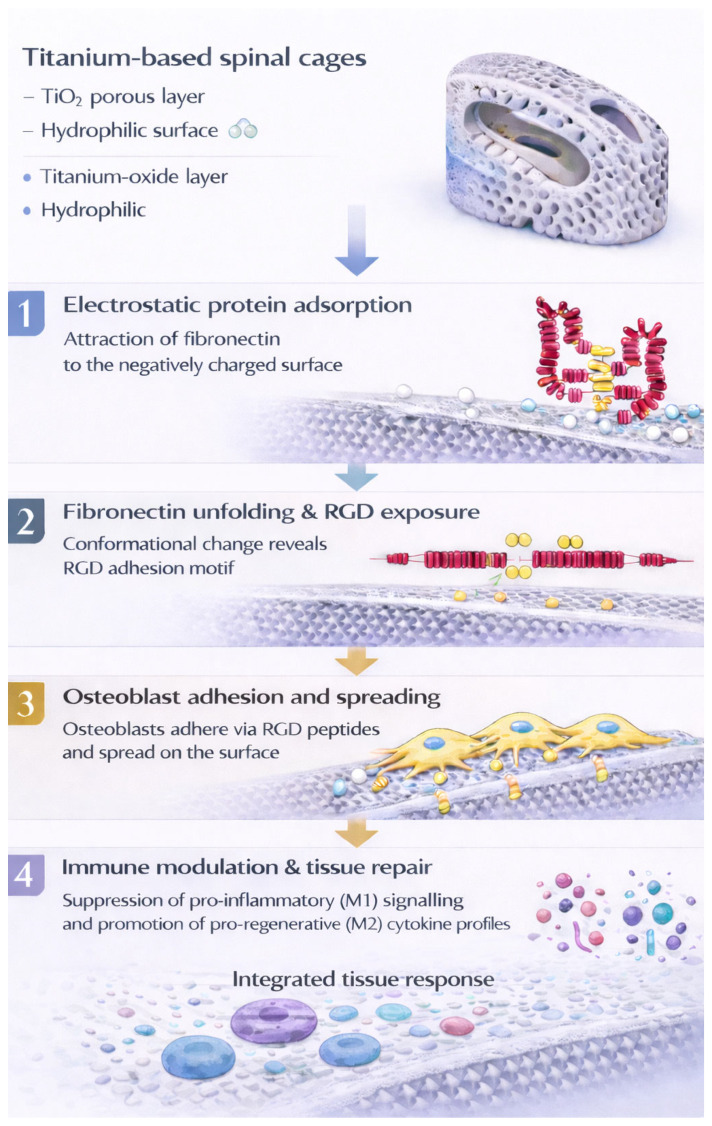
Schematic illustration of immune modulation by titanium oxide surfaces, highlighting protein-mediated processes associated with M2-like macrophage responses and suppression of pro-inflammatory signalling. The schematic represents simplified pathways and does not capture the full complexity or variability of immune responses, which are influenced by multiple context-dependent factors.

This section focuses specifically on compositional and surface-related effects, while structural and mechanical influences are addressed separately in subsequent sections. Material composition plays a central role in governing osseointegration in FGM spinal cages. Following implantation, material surfaces interact with the host environment through a series of processes, including protein adsorption, immune modulation, angiogenesis, and osteogenic differentiation [27,28]. In titanium-based implants, these processes are strongly influenced by the naturally formed titanium oxide layer. This layer exhibits high surface energy, corrosion resistance, and hydrophilicity, which collectively enhance protein adsorption and cell adhesion [27,29]. At physiological pH, the negatively charged oxide surface may promote fibronectin adsorption and induce conformational changes that expose RGD motifs, potentially enhancing integrin-mediated osteoblast attachment (Figure 2) [30,31]. Surface wettability also influences immune behaviour. In orthopaedic implant studies, hydrophilic titanium surfaces have been reported to promote anti-inflammatory, M2-like macrophage responses under specific experimental conditions. These surfaces have also been associated with reduced expression of markers linked to pro-inflammatory signalling. Such shifts have been correlated with increased secretion of reparative mediators, including IL-10 and Arg-1, which are commonly associated with tissue regeneration and stable osseointegration [32,33]. However, titanium alloy exhibits relatively poor wear resistance and may generate cytotoxic debris under physiological conditions [34,35]. This can trigger an opposing biological response, involving the release of pro-inflammatory cytokines, activation of RANK/RANKL signalling, differentiation of osteoclasts, and ultimately osteolysis [36]. Such contrasting effects highlight the need to balance bioactivity with long term material stability. To address the associated limitations, gradient material and coating strategies have been developed. These approaches aim to preserve the biocompatibility of titanium while improving mechanical performance and biological outcomes. The specific mechanisms and design strategies of these graded systems are discussed below.

In implants incorporating a titanium-titanium nitride (TiN) gradient, nitride coatings increase surface hardness. However, the coating does not fully resolve the limited wear resistance of titanium alloys, as chloride ions may disrupt passivation and compromise long-term stability [37]. A gradient nano-TiN coating with controlled nitrogen composition forms multilayer structures that significantly enhance hardness (≈50%), adhesion strength (≈40%), and wear resistance (≈600%), while reducing the friction coefficient (≈25%) compared to monolayer TiN [38]. Biological data specific to TiN-coated spinal cages remain limited. However, studies in dental and orthopaedic implants suggest that TiN surfaces may reduce inflammatory signalling and enhance extracellular matrix (ECM) deposition [39]. ECM deposition and mineralisation play key roles in osteoblast differentiation and osteointegration. In the canonical inflammatory pathway, TLR4 recognises lipopolysaccharide and signals through MyD88, leading to NF-κB p65 activation and subsequent NLRP3 inflammasome formation. This cascade may contribute to the expression of pro-inflammatory genes. TiN coatings have been hypothesised, primarily in dental and orthopaedic implant studies, to modulate the pathway, potentially through altered protein adsorption and receptor engagement. As a result, reduced inflammatory signalling could contribute, in certain contexts, to lower cytokine release and a local environment more conducive to tissue healing [39]. However, reported cellular responses to TiN remain variable and appear to depend on coating quality, surface characteristics, and cell type [40]. These inconsistencies highlight the need for further investigation, particularly in spine-specific applications. While these mechanistic pathways provide valuable insight into potential cell–material interactions, it is important to note that much of the available evidence is derived from orthopaedic implants, dental systems, and general biomaterial studies rather than spine-specific models. The direct applicability of these mechanisms to spinal cages remains to be fully established. Immune responses are highly context-dependent and influenced by local mechanical, biological, and anatomical factors. Accordingly, these responses should be interpreted within the context of the specific implant system and biological environment.

In implants with a composition gradient from titanium to hydroxyapatite (Ca_10_(OH)_2_(PO_4_)_6_; HA), the bioactivity is enhanced due to the chemical similarity of HA to native bone and strong osteoconductive properties [41,42]. HA degradation releases calcium ions that likely promote serum protein adsorption and formation of a biologically favourable protein layer at the implant surface, as widely reported in bone tissue engineering and orthopaedic implant studies [43,44]. Fibronectin–calcium phosphate complexes formed on HA surfaces enhance mesenchymal stem cell adhesion and osteogenic differentiation, as indicated by increased ALP activity, Runx2 expression, and BMP-2 signalling [45,46]. Collectively, the mentioned biological effects have been associated with activation of the MAPK pathway. Despite the well-established benefits of hydroxyapatite in enhancing spinal implant performance [47], conventional HA coatings often exhibit poor interfacial adhesion due to thermal expansion mismatch with titanium substrates [48]. To address these limitations, functionally graded HA coatings incorporating transition layers significantly improve adhesion strength (≈50%), thereby enhancing coating durability and reducing the impact of thermally induced stress [48]. Moreover, Thermal treatment can be used to control coating crystallinity, thereby regulating ion release, surface energy, and overall bioactivity [41,48]. Amorphous hydroxyapatite may promote osteogenic activity through increased ion release, whereas crystalline coatings improve long-term stability. Gradient-crystallinity designs aim to balance these effects, potentially enhancing osteogenic activity without compromising coating durability (Table 2) [49].

Another composition gradient is from titanium to poly(ether) ether ketone/polydimethylsiloxane (PEEK/PDMS). Beyond low wear resistance, titanium spinal implants (commonly Ti-6Al-4V) possess a significantly higher elastic modulus than human bone (110 GPa vs. 12 GPa) [50,51]. The mechanical mismatch induces uneven load distribution, stress shielding and subsidence, which may contribute to osteocyte apoptosis, increased RANKL signalling, subsequent bone resorption, and implant subsidence. PEEK (4GPa) [52] exhibits more similar mechanical properties to adjacent vertebral bone than titanium, making it more suitable for spinal fusion cage applications. However, its inherent hydrophobicity and low surface energy often limit protein adsorption and cellular adhesion [53,54,55]. To bridge this gap, Kishimoto et al. [56] designed and fabricated a novel functionally graded PEEK/PDMS porous spinal implant structure with a titanium oxide surface to minimize stress shielding while enhancing osseointegration. By incorporating gradual material transitions, this design aimed to minimise the risk of stress shielding and maximise osseointegration. However, the study lacks mechanical and biological testing and results.

### 4.2. Structural Gradient

This section examines geometric design parameters, including pore size, porosity, and lattice architecture, independent of compositional effects. In addition to surface chemistry, structural parameters such as porosity, pore size, and lattice structure strongly influence cellular responses to implant materials. Functional grading of these features allows stiffness, mass transport, and vascularisation to be tuned to support improved osseointegration [57,58].

#### 4.2.1. Effect of Pore Size

**Figure 3:**
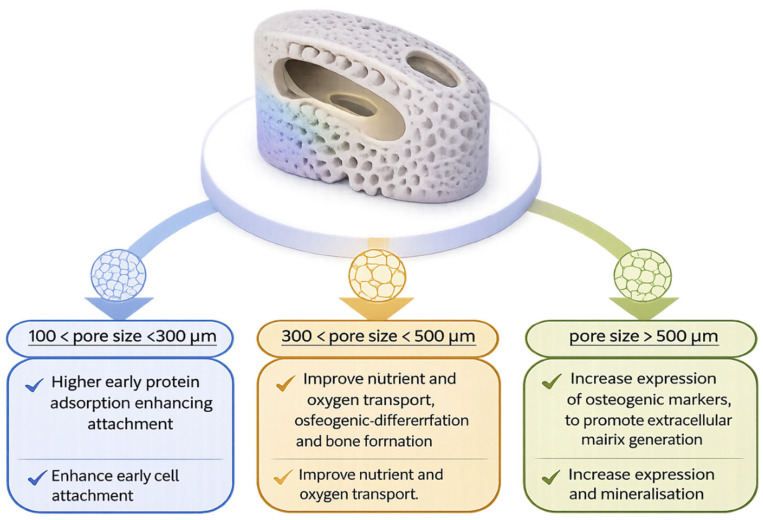
Effect of pore size gradients in functionally graded spinal cages on cell adhesion, bone formation, and implant stability. The relationships illustrated are simplified representations and do not fully capture the complex, context-dependent nature of biological responses to pore architecture.

Interconnected porosity in FGM spinal cages permits infiltration of cells and proteins throughout the implant volume, supporting internal bone formation and improving implant stability and osseointegration [59,60]. Pore size is widely reported to influence these responses in porous biomaterials (Figure 3) as it controls both cell–surface interactions and mass transport within the scaffold. Smaller pores (approximately 100–300 μm) favour early cell attachment due to increased surface area for protein adsorption, whereas intermediate pore sizes (300–500 μm) improve oxygen and nutrient diffusion and support osteogenic differentiation [61,62,63,64,65,66]. Larger pores (≥500 μm) have been associated, primarily in preclinical and scaffold studies, with increased bone volume fraction and elevated expression of osteogenic markers. Elevated expression of ALP, BMP-2, collagen type I (COL-1), and Runx2 may reflect enhanced matrix mineralisation and osteoblast maturation [61]. Conversely, smaller pores offer greater surface area for protein adsorption, which enhances cell attachment and promotes early osseointegration [66]. Collectively, these complementary effects may explain why single-pore designs do not fully address both early and long-term biological requirements. Functionally graded pore architectures address this limitation by spatially combining pore sizes with distinct biological roles. In vitro studies consistently show that smaller pores likely promote early cell adhesion, while larger pores favour osteogenic differentiation and matrix production. For example, polycaprolactone (PCL) scaffolds with graded pores (250–750 μm) exhibited higher ALP and osteocalcin (OCN) activity in larger pores, whereas smaller pores preferentially supported early cell attachment [67]. ALP may facilitate initial osteoblast differentiation and mineralisation, whereas OCN regulates matrix mineralisation and bone organisation, together accelerating the formation of a functional bone matrix [67]. Similar trends were observed in PEOT/PBT scaffolds with pore sizes ranging from 500 to 1100 μm. Larger pore size was associated with increased ALP expression, whereas smaller pores exhibited greater calcium deposition, indicating early mineralisation [68]. Taken together, the available evidence suggests that graded pore designs may help balance early implant fixation with long-term bone remodelling. Smaller pores are thought to support early implant stability through more rapid initial bone formation. Larger pores likely facilitate vascularisation, nutrient exchange, and sustained bone ingrowth over time, supporting the use of graded porosity as a potential strategy for optimising spinal implant performance.

While these trends are widely reported, the relationship between pore size and biological outcomes is not uniform across studies [58,59,60,61,62,63,64,65]. Reported responses may vary depending on material composition, surface properties, manufacturing method, and experimental conditions. For example, pore sizes associated with enhanced osteogenic differentiation in one system may not produce equivalent outcomes in another, highlighting the context-dependent nature of cell–material interactions [65,66]. In addition, pore size optimisation involves inherent trade-offs between biological performance and mechanical stability [12]. Larger pores may facilitate vascularisation and bone ingrowth but can also reduce structural integrity and fatigue resistance. In contrast, smaller pores may enhance early cell attachment and increase the surface area available for protein adsorption, although they likely restrict nutrient transport and tissue infiltration. Collectively, these competing effects suggest that no single pore size is universally optimal and that graded or spatially varied designs can provide a more balanced approach.

It should be noted that many of these findings are derived from scaffold-based or non-spinal models, and their direct applicability to spinal fusion cages may be influenced by differences in anatomical environment, loading conditions, and clinical context. Furthermore, the optimal pore architecture may depend on patient-specific factors, including bone quality, age, and metabolic status, as well as surgical considerations such as insertion technique and load-bearing requirements.

#### 4.2.2. Effect of Porosity

Functionally graded porosity is designed to more closely reproduce physiological stress distributions at the bone–implant interface, thereby reducing stress shielding and supporting osseointegration [58,69]. Appropriate load transfer is critical for initiating normal bone remodelling. Mechanical strain has been reported to activate mechanotransduction pathways in osteoblasts, involving integrins and cytoskeletal networks. This has been associated with downstream osteogenic signalling, including Runx2 expression, with focal adhesion kinase pathways implicated in the process [45,70]. When implants are excessively stiff, they can bear a disproportionate share of the load, leaving adjacent bone under-stimulated and unable to maintain normal remodelling activity. This phenomenon, referred to as stress shielding, may promote bone resorption and progressive structural weakening [71,72]. Introducing porosity gradients helps align implant stiffness more closely with that of vertebral bone, mitigating these effects while maintaining sufficient mechanical support.

#### 4.2.3. Mechanical Implications of Porosity

Studies have demonstrated that graded porosity can improve both mechanical behaviour and biological response in spinal implants. For example, Ti-6Al-4V lumbar cages with longitudinal porosity gradients distribute strain more evenly across the vertebral endplate than uniform designs, reducing stress shielding and enhancing load transfer [17,73,74]. Reducing the effective Young’s modulus toward physiological values allows graded designs to preserve mechanotransductive signalling, potentially promoting adaptive bone remodelling. In contrast, uniformly high-porosity implants could compromise mechanical integrity and increase the risk of instability [74,75,76]. Beyond mechanical effects, porosity gradients shape cellular behaviour by supporting early cell attachment in smaller pores and promoting osteogenic differentiation, vascularisation, and nutrient transport in larger pores. By combining these functions, graded scaffolds enhance osseointegration while reproducing key features of bone architecture [67,68,71,72,74].

#### 4.2.4. Effect of Lattice Structure

**Figure 4:**
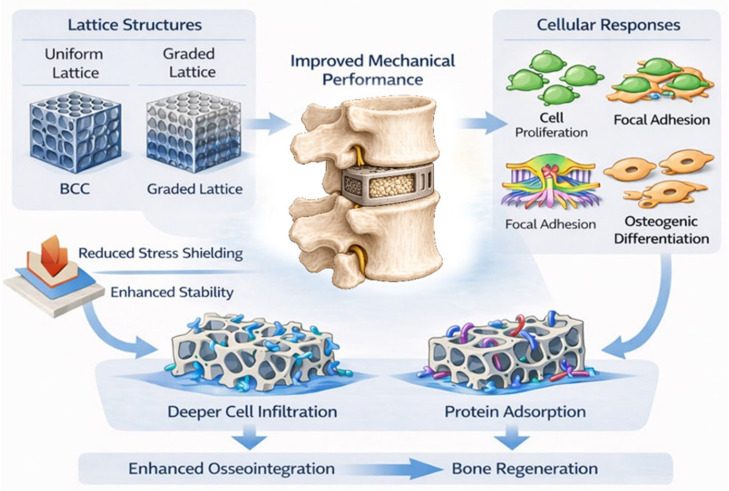
Schematic overview of the influence of lattice architecture on mechanical load transfer and biological responses in spinal implants. Structural gradients promote cell infiltration, protein adsorption, and osteogenic differentiation while reducing stress shielding.

Lattice architecture is known to influence both the mechanical behaviour and biological performance of porous orthopaedic implants. Key design variables, including unit-cell orientation and connectivity, strut thickness, and unit size, govern how loads are transferred through the implant and how cells interact with its surface [70,77,78]. Among commonly used lattice types, such as body-centred cubic (BCC), face-centred cubic, and diamond structures, BCC lattices are more flexible and exhibit a more uniform stress distribution. Their high porosity can reduce stress shielding while supporting cell adhesion [71]. However, uniform lattice designs inherently restrict spatial variation in pore size and porosity and implicitly assume homogeneous vertebral mechanics, which can limit their ability to support optimal osseointegration. Functionally graded lattice designs overcome these limitations by introducing spatial variations in architecture that better reflect the mechanical and biological heterogeneity of bone. Experimental studies have shown that octet-truss, tetrahedral, and hybrid octet-truss–tetrahedral lattices with longitudinal porosity gradients (60–80%) promote higher cell proliferation than uniform counterparts [51,77,78,79]. Similarly, gradient rhombus lattices with progressively changing strut angles (23° to 36°) and pore sizes (200 to 600 μm) have been shown to enhance focal adhesion formation, actin and vinculin expression, and integrin-mediated mechanotransduction [51,78,79,80]. These architectural features have been shown to promote deeper cell infiltration and more controlled protein adsorption, which may contribute to improved osteogenic signalling [79,81,82] (Figure 4).

#### 4.2.5. Mechanical Implications of Lattice Structure

Evidence from in vivo studies further supports the biomechanical relevance of graded lattice architectures. For example, Voronoi-tessellation lattices with gradually increasing strut thickness produce corresponding gradients in porosity and pore size, yielding effective elastic moduli (approximately 600–1500 MPa) comparable to human bone and higher bone volume fractions than uniform structures after 12 weeks [56]. These findings demonstrate that lattice gradients can be used to tune mechanical compliance while enhancing bone formation. At the cellular level, increased expression of actin and vinculin observed in larger unit-cell regions reflects their central role in focal adhesion dynamics. Actin provides the cytoskeletal framework required for cell migration and attachment, while vinculin acts as a mechanosensitive linker that stabilises integrin–talin complexes under load [83,84,85]. These findings illustrate how controlled lattice grading can enable simultaneous optimisation of mechanical performance and cell-mediated bone regeneration. The key relationships between lattice parameters, biological pathways, and functional outcomes are summarised in Table 3.

### 4.3. Manufacturing Techniques

Recent advances in additive manufacturing (AM) now enable the fabrication of functionally graded spinal cages from metals, polymers, and bioactive composites. The layer-by-layer nature of AM offers fine spatial control, making it particularly suitable for introducing structural gradients and tailoring mechanical properties [87]. Achieving smooth transitions within the implant, however, depends on careful optimisation of processing parameters. AM approaches for FGMs are commonly categorised into structural gradient methods, coating-based gradient techniques, and nanoscale fabrication strategies.

#### 4.3.1. Structural Gradient

Structural gradients in pore size, porosity, and lattice geometry are usually defined at the CAD stage and subsequently realised through additive manufacturing. Selective laser melting (SLM) enables the fabrication of high-resolution metallic lattices with feature sizes of approximately 70–120 μm, while electron beam melting (EBM) produces structures with lower resolution (100–500 μm) but improved ductility and wear resistance [77,80,87,88,89,90,91,92]. Selective laser sintering (SLS) is commonly used for polymers and polymer–ceramic composites, such as PCL–HA and PEEK–βTCP, allowing bioactive components to be retained during processing [93,94].

**Figure 5:**
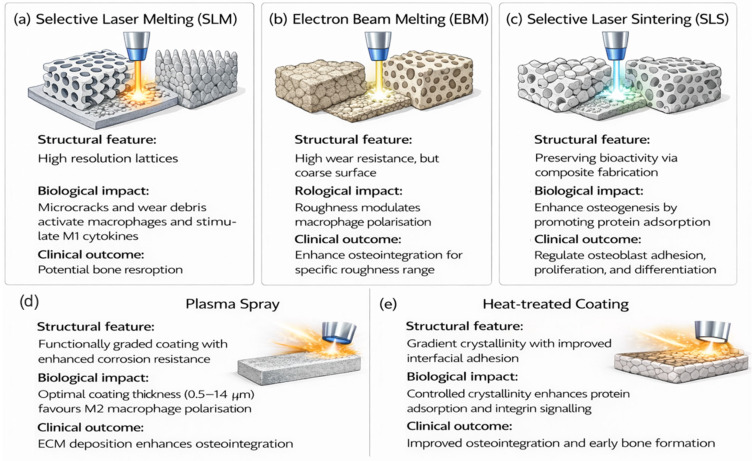
Additive manufacturing and surface modification strategies for spinal implants and their effects on structure, host immune response, and osseointegration. (**a**) SLM, (**b**) EBM, and (**c**) SLS control lattice architecture, surface roughness, and bioactivity, influencing macrophage polarisation and osteogenesis. (**d**) Plasma spray coatings and (**e**) heat-treated coatings modulate interfacial properties to enhance protein adsorption, immune regulation, and bone formation. This figure presents a conceptual integration of processing–structure–biology relationships. These links represent associations reported in the literature and do not imply direct or universal causality.

In addition to geometric control, these manufacturing routes also influence surface morphology and microstructure, which may affect biological response. SLM-fabricated surfaces typically exhibit moderate roughness (5–40 μm) and α′ martensitic microstructures that have been reported to generate microcracks and wear debris [80,87,88]. Microcracks and wear debris have been associated with macrophage activation and increased expression of M1-associated cytokines, including TNF-α, IL-1β, IL-6 and RANKL-mediated osteoclast activation (Figure 5a) [36]. By comparison, EBM produces coarser surfaces (25–135 μm) and more stable α/β lamellar microstructures, which may reduce debris formation and support improved osseointegration [95,96,97,98,99,100].

Surface roughness may also influence immune behaviour. Intermediate roughness values (approximately 0.5–1.4 μm) have been reported to be associated with anti-inflammatory, M2-like macrophage polarisation. However, deviations from this range have been linked, in some studies, to increased pro-inflammatory signalling and impaired bone integration (Figure 5b) [101,102]. For polymer-based systems, SLS-fabricated bioactive composites have been reported to promote osteogenesis, potentially through enhanced adsorption of proteins such as fibronectin and vitronectin. This process may contribute to the activation of integrin-mediated signalling pathways, including ERK (extracellular signal-regulated kinase) and Wnt (Wingless-related integration site), which are involved in regulating stem cell and osteoblast adhesion, proliferation, and differentiation (Figure 5c) [45,103,104,105,106]. Comparable trends have been observed in magnesium-containing scaffolds, where increased integrin expression has been associated with enhanced focal adhesion, cytoskeletal organisation, and mechanotransduction [103]. While surface roughness has been associated with changes in macrophage behaviour, reported trends vary across studies and depend on factors such as roughness scale, surface chemistry, manufacturing route, and cell type. As a result, the relationship between roughness and macrophage polarisation should therefore be interpreted as context-dependent rather than universal.

#### 4.3.2. Coating Gradient

Functionally graded coatings, particularly plasma-sprayed or thermally treated HA, are used to improve both interfacial stability and biological performance (Figure 5d,e). Coating thickness is a key factor in achieving this balance. Thick HA layers (up to ~220 μm) provide a protective barrier that limits corrosion and reduces the release of potentially harmful ions from the titanium substrate [48]. In contrast, thinner coatings (0.5–1.4 μm) have been reported to exert a stronger influence on immune responses. A few studies have associated thin coatings with anti-inflammatory, M2 macrophage polarisation and reduced pro-inflammatory signalling, which may support osteogenesis and early osseointegration [48,101,107,108,109,110,111,112].

Plasma spraying enables the deposition of relatively thick coatings, typically in the micrometre range, although submicron features may also be present depending on processing conditions [109,110]. Following implantation, the coating surface rapidly adsorbs proteins such as albumin and fibronectin, which likely contribute to macrophage recruitment. Excessive M1 activation has been associated with sustained inflammatory signalling and possibly accelerates coating degradation through the formation of an acidic microenvironment. Conversely, coatings within the optimal thickness range (0.5–1.4 μm) have been associated with macrophage responses consistent with M2-like phenotypes and a local environment that may favour osteoblast recruitment and ECM formation [101,111,112]. Beyond immune modulation, HA coatings likely enhance corrosion resistance and provide a roughened surface that supports cell adhesion and proliferation. When implemented as FGM systems, these coatings likely integrate mechanical protection with biological stimulation. This combination has been suggested to influence integrin-mediated signalling associated with osteogenesis and osseointegration [48,108]. Much of the available evidence is derived from orthopaedic and in vitro studies; therefore, direct translation to spinal implants should be interpreted with caution.

It is important to note that the functional performance of gradient coatings is closely related to their degradation behaviour, interfacial stability, and capacity for controlled release of bioactive agents. Sequential degradation of multilayer or graded coatings has been proposed as a strategy to achieve time-dependent biological responses, where outer layers may provide early-stage bioactivity while inner layers contribute to long-term stability. Interfacial bonding between coating layers and the underlying substrate is also critical, as weak adhesion may lead to delamination under cyclic loading, thereby affecting both mechanical integrity and biological performance. Furthermore, gradient coatings have been explored as platforms for controlled release of therapeutic agents, including ions, growth factors, and antimicrobial compounds. These systems likely enable spatial and temporal regulation of biological responses, although the release kinetics are influenced by coating composition, microstructure, and degradation rate. Recent studies have highlighted the importance of tailoring gradient architectures to achieve coordinated mechanical support, degradation behaviour, and biological functionality [113,114]. Despite these advances, the translation of such strategies to spinal applications remains limited, and further investigation is required to understand their long-term performance under physiologically relevant conditions.

Manufacturing techniques shape the structural architecture, surface characteristics, microstructure, and coating properties of spinal implants. These factors influence cell adhesion, immune behaviour, osteogenic signalling, and mechanotransduction, thereby influencing the biological performance of functionally graded spinal cages (Table 4).

Overall, while additive manufacturing and surface modification strategies enable precise control of implant architecture and surface characteristics, the relationships between processing parameters, microstructure, and biological response remain complex and not fully resolved. Reported outcomes vary across material systems, manufacturing conditions, and experimental models, and direct causal links are often difficult to establish. These relationships should be interpreted as indicative trends rather than definitive mechanisms, particularly in the context of spinal applications. Beyond these factors, additional biological considerations may arise in spine-specific applications. In addition to osseointegration and mechanical performance, the proximity of spinal implants to neural structures introduces additional considerations related to neurobiological safety [36]. Spinal implants are placed adjacent to the spinal cord and nerve roots, where local inflammatory responses, wear debris, or mechanical irritation may influence neural tissues. While current studies predominantly focus on bone integration and structural performance, the potential interaction between FGM implant features and the neural environment remains largely underexplored. Factors such as material degradation, ion release, and micro-motion at the implant interface may contribute to local biological responses that extend beyond bone tissue. Clinical investigations of periprosthetic tissues and in vitro studies have revealed that wear debris from spinal implants can also reduce astrocyte and microglial cell viability in the spinal cord [115,116,117]. Although direct evidence remains limited, these considerations highlight the importance of incorporating neurobiological safety into future design and evaluation frameworks for functionally graded spinal implants.

## 5. Research Gaps and Challenges

Despite increasing research activity, several key challenges continue to limit the clinical translation of functionally graded spinal cages. These challenges can be broadly grouped into biological, methodological, translational, and mechanical validation gaps. Together, they highlight critical areas where further investigation is required to support the development of more reliable and clinically effective implant designs.

From a biological perspective, current studies predominantly focus on osteogenic responses, including osteoblast activity, cell adhesion, and expression of markers such as alkaline phosphatase (ALP), often in conjunction with assessments of pore-driven permeability and bone ingrowth [59,60,61,62,63,64,65,66]. However, angiogenic signalling and macrophage-mediated immune responses, both essential regulators of successful fusion, remain comparatively unexplored, particularly in spine-specific studies of FGM implants. Angiogenesis, largely regulated by VEGF, plays a central role in linking vascularisation with osteogenesis, yet direct quantification of angiogenic markers remains uncommon in studies of FGM spinal cages [118,119,120,121,122].

From a methodological perspective, much of the available evidence is derived from in vitro systems, scaffold-based models, and orthopaedic implants. While these models provide valuable mechanistic insights, they do not fully replicate the complex physiological and mechanical environment of the spine. Mechanical behaviour is frequently inferred from finite element modelling, with stress distribution and stress shielding often used as indirect proxies for biological integration. Direct experimental assessment of mechanotransduction (particularly integrin- and cytoskeleton-mediated signalling leading to activation of osteogenic regulators such as Runx2) remains technically challenging and is therefore rarely incorporated into experimental study design [123,124,125,126,127].

From a translational perspective, interpretation and comparison across studies are complicated by a lack of standardisation in experimental design and reporting. Many studies evaluate isolated biological outcomes without fully considering trade-offs between competing processes, such as osteogenesis and inflammation. For example, large pore sizes may enhance bone formation, as reported in scaffold and preclinical studies [68], yet excessive porosity has also been associated with increased inflammatory responses in biomaterial and implant studies [92]. Surface properties, including roughness, wettability, and surface energy, are often insufficiently characterised, despite their strong influence on immune and cellular behaviour. These factors may also be unintentionally altered when gradients are introduced, further complicating interpretation [102].

Clinically relevant degradation mechanisms represent an additional translational gap. Wear, corrosion, and debris generation under physiological loading conditions have been shown in orthopaedic and biomaterial studies to activate inflammatory pathways and promote osteolysis [36,111]. While increased porosity improves cell infiltration, it can also increase exposure to corrosive environments. Highly porous implants have been reported to show greater susceptibility to pitting corrosion due to electrolyte penetration [128]. However, these degradation-related effects are rarely incorporated into existing graded design frameworks.

An additional and often overlooked consideration is the anatomical proximity of spinal implants to the spinal cord and surrounding neural structures. While most studies focus on osseointegration and mechanical performance [129], the potential impact of graded design features on neural tissues, including risks associated with inflammatory mediators, wear debris, or local mechanical irritation, remains insufficiently explored [117,130]. Given the confined anatomical environment of the spinal canal, even subtle changes in implant behaviour may have clinically significant consequences. This highlights the need for more comprehensive neuro-biological safety assessments.

Emerging experimental platforms may help address some of the limitations. Current models remain limited in their ability to replicate the complex physiological environment of the intervertebral disc and surrounding tissues. Disc-on-a-chip [131] and organ-on-a-chip systems [132,133,134] offer promising opportunities to study coupled mechanical, biological, and biochemical responses under more physiologically relevant conditions. Organ models enable controlled investigation of cell–material interactions, nutrient gradients, and dynamic loading, potentially bridging the gap between simplified in vitro assays and in vivo studies.

Finally, comprehensive mechanical validation of functionally graded designs remains limited. Many studies, particularly in computational and preclinical settings, emphasise stiffness derived from geometry, while fatigue resistance, ductility, wear resistance, and long-term durability under multiaxial loading are often neglected [135,136]. Although established testing standards (ASTM F1717, ASTM F2077, ISO 12189) exist, they are infrequently applied to functionally graded designs, particularly in spine-specific contexts [137,138,139].

These gaps have direct implications for the proposed design-to-biology framework. In particular, the limited integration of immune and vascular responses, the reliance on simplified experimental models, and the lack of long-term mechanical validation constrain the ability to establish robust links between graded design features and biological outcomes. Addressing these challenges will be essential for refining the framework and improving its predictive and translational relevance. While mechanistic hypotheses are increasingly supported by in vitro and preclinical studies, definitive causal relationships between specific graded design features and long-term clinical fusion outcomes in spinal applications have yet to be fully established.

## 6. Clinical Implications for Spinal Fusion Practice

The clinical implications of FGM spinal cages remain incompletely defined due to the limited availability of direct clinical evidence. Most current insights are derived from preclinical, computational, and in vitro studies, and therefore should be interpreted with caution when considering their applicability to patient outcomes.

Rather than representing a validated clinical solution, FGM spinal cages can be viewed as a design approach that introduces additional parameters for tailoring implant behaviour. These parameters, including gradients in stiffness, porosity, and surface bioactivity, may influence load transfer, bone ingrowth, and local biological responses. However, the extent to which these effects translate into improved fusion rates, reduced complications, or better long-term outcomes in patients has not yet been established.

From a surgical perspective, stiffness gradients may be relevant in cases where bone quality is compromised, such as in osteoporotic patients [14,15,16,17,18]. Gradual transitions in mechanical properties can support distributed load transfer, although the optimal gradient profile is likely to depend on patient-specific factors and surgical technique. At the same time, increased porosity or structural complexity may introduce practical challenges, including reduced insertion strength or increased susceptibility to endplate damage during implantation.

Similarly, graded porosity and lattice architectures may influence biological processes over time, but their effects are not uniform. Designs that combine smaller pores at the implant–bone interface with larger internal pores have been proposed to balance early stability with longer-term bone ingrowth [67,68,80]. However, such strategies must be considered in the context of patient variability, including differences in healing capacity, metabolic conditions, and inflammatory status.

Surface and material gradients introduce further complexity. Bioactive coatings and compositional gradients can support early osseointegration, but their long-term behaviour under cyclic spinal loading remains uncertain [45,46,47]. Issues such as wear, corrosion, and immune-mediated responses likely influence implant performance over time, particularly in mechanically demanding environments [36]. In addition, imaging and postoperative assessment may be affected by graded material compositions and internal architectures [25]. While some material combinations may improve radiographic visibility, heterogeneous structures may complicate interpretation of fusion progression.

Overall, FGM spinal cages should be considered as an emerging design strategy rather than an established clinical solution. Their potential clinical value will depend on future studies that directly evaluate their performance in vivo, including controlled clinical trials and long-term outcome assessments. At present, their role in spinal fusion practice remains exploratory, and careful consideration of both their potential advantages and uncertainties is required.

## 7. Conclusions

FGM spinal cages represent an evolving approach that integrates material design and structural optimisation to address limitations associated with conventional interbody implants. By enabling spatial variation in composition, porosity, and mechanical properties, these designs aim to better align implant behaviour with the biological and mechanical environment of the spine. However, despite promising findings from preclinical and biomaterial studies, the current evidence base remains limited. Much of the available data is derived from in vitro systems, scaffold-based models, and non-spinal applications, and therefore may not fully capture the complexity of spinal fusion. Key aspects such as immune response, angiogenesis, long-term degradation, and mechanical durability under physiological loading conditions remain insufficiently understood. As a result, the biological and clinical implications of functionally graded spinal cages should be interpreted with caution. While these designs offer potential advantages, definitive evidence linking specific graded features to improved fusion outcomes or patient benefit is not yet established. Future progress will depend on the integration of materials science, mechanobiology, and clinical research. This includes the development of experimental models that better replicate the spinal environment, systematic evaluation of biological and mechanical trade-offs, and the application of standardised testing frameworks. Importantly, well-designed in vivo studies and clinical trials will be required to validate the translational relevance of graded implant strategies. FGM spinal cages should be viewed as a promising but still developing design paradigm, whose clinical value remains to be established through further rigorous investigation.

## Figures and Tables

**Table 1:** Classification framework for FGM spinal cages.

Category	Example Features
Material Gradient	Metal–ceramic, Polymer–ceramic (e.g., Ti–HA, PEEK–βTCP) [23]
Structure Gradient	Gradient porosity or structure to mimic bone structure [24]
Mechanical Gradient	Tailored mechanical properties to match vertebral bone [13,14]
Biological Function	Surface optimisation to enhance cell responses [25]
Manufacturing Method	Additive manufacturing, powder metallurgy, and coating [8]

HA: Hydroxyapatite, PEEK–βTCP: Poly(ether ether ketone)–beta tricalcium phosphate composite.

**Table 2:** Surface features of material- and composition-graded spinal cages and the biological pathways supporting improved biological performance. Biological pathways were derived from orthopaedic implants, dental systems, and general biomaterial studies rather than spine-specific models. Cellular responses may vary based on coating quality, surface characteristics, and cell type.

**Traditional spinal cages**		
**Material composition**	Surface characteristics	Biological pathway
**Titanium oxide**	High surface energy Hydrophobic surface Low wear resistance	Enhancing osteoblast cell attachment via fibronectin attraction [27,29]
**Material- and composition-graded spinal cages**		
**Gradient nano-TiN coating**	Superior nano-hardness Improved wear resistance Low long-term stability of surface passive layer	Suppressing the activation of NLRP3 inflammatory pathway [39,40]
**HA coating**	Superior biological properties Weak coating stability	Activation of MAPK pathway supporting osteoblast attachment [41,48]
**Graded HA coating**	Improved coating stability Weak biological performance at higher crystallinity	

**Table 3:** Influence of scaffold structural parameters on biological outcomes and osteogenic signalling pathways relevant to bone tissue integration.

Structural Parameter: **Pore Size**	
Key feature	Interconnected pores; small (100–300 μm) vs. large (~500 μm); gradient 250–1100 μm [67,68]
Biological outcomes	Small pores: early cell adhesion and initial bone formation; Large pores: enhanced vascularisation, osteoblast differentiation, and matrix mineralisation; Gradient pores: support both early and long-term bone integration [61,62,63,64,65,66,67,68]
Biological pathways	Small pores enhance protein adsorption, which may promote integrin-mediated cell adhesion; large pores improve nutrient and oxygen transport, stimulating osteogenic differentiation through markers such as ALP, OCN, BMP-2, COL-1, and Runx2 [61,62,63,64,65,66,67,68]
Limitations	Reported effects are context-dependent and may vary with material type, surface properties, and experimental conditions. Larger pores may enhance vascularisation but can reduce mechanical strength, while smaller pores improve initial adhesion but may limit nutrient transport.
Structural parameter: **Porosity**	
Key feature	Low vs. high porosity; longitudinal gradients in spinal cages; Young’s modulus tailored to bone [58,69,74]
Biological outcomes	Small pores/low porosity: early cell adhesion; Large pores/high porosity: osteogenic differentiation, nutrient/waste exchange, and vascularisation; Gradient porosity: adaptive mechanical support and improved osseointegration [17,70,71,72,73,74]
Biological pathways	Load-induced strain is reported to activate mechanotransduction in osteoblasts through integrins and cytoskeletal networks. This has been associated with downstream osteogenic signalling, including upregulation of Runx2 expression via focal adhesion kinase pathway [67,68,71,72]
Limitations	High porosity may improve biological integration but can compromise mechanical integrity and increase susceptibility to deformation or failure. Conversely, low porosity enhances structural stability but may restrict cell infiltration and mass transport.
Structural parameter: **Lattice structure**	
Key feature	Unit cell type (BCC, FCC, diamond), strut thickness, pore size, porosity; graded lattice designs (e.g., octet-truss, rhombus, Voronoi) [70,77,78]
Biological outcomes	Increased cell proliferation, adhesion, and migration; improved osteogenic signalling; biomimetic stress distribution; higher bone volume fraction [51,75,77,78,79,80,81,82,86]
Biological pathways	Integrin-mediated mechanotransduction may promote actin cytoskeleton and vinculin expression, enhance focal adhesion formation, and regulate protein adsorption to support osteogenic signalling [83,84,85]
Limitations	Biological and mechanical responses depend on unit-cell geometry, manufacturing accuracy, and loading conditions. Uniform lattice designs may not capture local mechanical variation, while highly complex graded structures may introduce fabrication challenges and variability in performance.

Reported biological outcomes reflect trends observed across heterogeneous in vitro and preclinical studies. These relationships are not uniform and may vary with material system, manufacturing parameters, and experimental context, and therefore should be interpreted as indicative rather than definitive.

**Table 4:** Summary of the fabrication method and its pros and cons.

Fabrication Method	Key Features
Selective Laser Melting (SLM)	High-resolution metallic lattices (spot size 70–120 μm), α′ martensite microstructure, surface roughness 5–40 μm [80,87,88]
	Biological pathways (Mechanism)
	Microcracks and wear debris may activate macrophages, stimulating M1 cytokines (TNF-α, IL-1β, IL-6) and RANKL-mediated osteoclast activation, which lead to bone resorption [36,95,96,97,98,99,100,101,102]
	Biological outcomes
	Potential bone resorption if excessive debris is present
	Limitations
	Microcracks, debris generation, variability in surface chemistry
Electron Beam Melting (EBM)	Coarse surfaces (roughness: 25–135 μm), α/β lamellae, higher ductility and wear resistance [88,91,95,97,98,99,100]
	Biological pathways (Mechanism)
	Reduced debris promotes osteointegration. Roughness may influence macrophage polarisation, with a roughness range of 0.5 < roughness < 1.4 favouring anti-inflammatory M2 polarisation [95,96,97,98,99,100]
	Biological outcomes
	Reduced inflammation, favouring osteointegration for specific roughness ranges.
	Limitations
	Lower resolution, surface coarseness variability
Selective Laser Sintering (SLS)	Allows fabrication of composites, preserving bioactive properties [45,103,104,105,106]
	Biological pathways (Mechanism)
	Protein adsorption (fibronectin, vitronectin) activates integrin-mediated signalling, including ERK and Wnt pathways, regulating stem cell and osteoblast adhesion, proliferation, and differentiation [45,46,103,105,106]
	Biological outcomes
	Enhanced osteogenesis, cell attachment, and ECM deposition; improved focal adhesion and actin cytoskeleton organisation.
	Limitations
	Polymer degradation, lower mechanical strength
Plasma-sprayed HA coatings	Allow functionally graded coatings (thickness up to 220 μm), enhance corrosion resistance [48].
	Biological pathways (Mechanism)
	Optimal coating thickness (0.5–1.4 μm) favours M2 macrophage polarisation, which secretes anti-inflammatory cytokines and pro-regenerative signals [48,101,107,108,109,110,111,112]
	Biological outcomes
	Promote osteoblast recruitment and extracellular matrix deposition
	Limitations
	Adhesion durability, long-term stability, delamination risk
Heat-treated coatings	Gradient crystallinity, improved interfacial adhesion [48,108]
	Biological pathways (Mechanism)
	Controlled crystallinity enhances protein adsorption and integrin signalling [48,108]
	Biological outcomes
	Improved osseointegration and early bone formation
	Limitations
	Lower biological performance in higher crystallinity

Reported biological outcomes reflect trends observed across heterogeneous in vitro and preclinical studies; responses may vary with material system, manufacturing parameters, and experimental conditions.

## Data Availability

No new data were created or analysed in this study. Data sharing does not apply to this article.

## Abbreviations

Abbreviation	Full Term/Definition
FGM	Functionally Graded Material
HA	Hydroxyapatite
PEEK	Poly(ether ether ketone)
PEEK-βTCP	Poly(ether ether ketone)–beta tricalcium phosphate composite
SLM	Selective Laser Melting
EBM	Electron Beam Melting
Ti-6Al-4V	Titanium–6 aluminium–4 vanadium alloy
M1	Classically activated (pro-inflammatory) macrophage phenotype
M2	Alternatively activated (anti-inflammatory) macrophage phenotype
IL-10	Interleukin-10
Arg-1	Arginase-1
NLRP3	NOD-, LRR-, and pyrin domain-containing protein 3
TLR4	Toll-like receptor 4
MyD88	Myeloid differentiation primary response 88
NF-κB p65	Nuclear factor kappa-light-chain-enhancer of activated B cells, p65 subunit
ALP	Alkaline phosphatase
BMP-2	Bone morphogenetic protein-2
MAPK	Mitogen-activated protein kinase
COL-1	Collagen type I
Runx2	Runt-related transcription factor 2
ECM	Extracellular matrix
PCL	Polycaprolactone
OCN	Osteocalcin
FAK	Focal adhesion kinase
BCC	Body-centred cubic
FCC	Face-centred cubic
AM	Additive manufacturing
TNF-α	Tumour necrosis factor-alpha
RANKL	Receptor activator of nuclear factor κB ligand
ERK	Extracellular signal-regulated kinase
Wnt	Wingless-related integration site signalling pathway
FGF-2	Fibroblast growth factor-2
VEGF	Vascular endothelial growth factor
ELISA	Enzyme-linked immunosorbent assay
PEOT/PBT	poly(ethylene oxide terephthalate)/poly(butylene terephthalate)
